# The Role of Glial Fibrillary Acidic Protein in Mediating and Moderating Alzheimer's Disease Pathologies

**DOI:** 10.1002/alz70856_099563

**Published:** 2025-12-24

**Authors:** Jun Sung Kim, Dahyun Yi, Min Soo Byun, Hyejin Ahn, Gijung Jung, Hyeji Choi, Jun‐Young Lee, Koung Mi Kang, Chul‐Ho Sohn, Yun‐Sang Lee, Yu Kyeong Kim, Dong Young Lee, KBASE Research Group

**Affiliations:** ^1^ Institute of Human Behavioral Medicine, Seoul National University Medical Reserach Center, Seoul, Seoul, Korea, Republic of (South); ^2^ Institute of Human Behavioral Medicine, Medical Research Center, Seoul National University, Seoul, Korea, Republic of (South); ^3^ Department of Neuropsychiatry, Seoul National University Hospital, Seoul, Korea, Republic of (South); ^4^ Interdisciplinary program of cognitive science, Seoul National University, Seoul, Korea, Republic of (South); ^5^ Department of Neuropsychiatry, Seoul National University Hospital, Seoul, Korea, Republic of (South); ^6^ Seoul National University Hospital, Seoul, Korea, Republic of (South); ^7^ Department of Psychiatry, Seoul National University College of Medicine, Seoul, Korea, Republic of (South); ^8^ Department of Radiology, Seoul National University Hospital, Seoul, Korea, Republic of (South); ^9^ Department of Nuclear Medicine, Seoul National University College of Medicine, Seoul, Korea, Republic of (South); ^10^ Department of Nuclear Medicine, SMG‐SNU Boramae Medical Center, Seoul, Korea, Republic of (South)

## Abstract

**Background:**

Glial fibrillary acidic protein (GFAP) is a major protein found in the astrocytic cells of the brain, and it is considered a marker of astrogliosis. This study aimed to examine whether blood GFAP levels moderate or mediate the relationships between in vivo Alzheimer's disease (AD) pathologies including beta‐amyloid (Aβ), tau, and neurodegeneration.

**Methods:**

A total of 92 older individuals with diverse cognitive spectrum were recruited from the Korean Brain Aging Study for the Early Diagnosis and Prediction of Alzheimer's disease (KBASE). All participants underwent [11C] Pittsburgh compound B‐PET, [18F] AV1451‐PET, [18F] FDG‐PET, and MRI. Plasma GFAP was measured using commercial Neurology 4‐plex E kit. We analyzed mediation and moderation effects of GFAP on the relationships between Aβ), tau, and neurodegeneration using the PROCESS Macro models 4 and 1 in SPSS. All analyses were adjusted for the effects of age, sex and apolipoprotein ε4 allele positivity.

**Results:**

A total of 92 participants (38 cognitive normal, 29 mild cognitive impairment and 25 AD dementia) were included. Plasma GFAP levels were significantly associated with all AD biomarkers: brain Aβ and tau deposition, AD‐signature cerebral glucose metabolism (AD‐CM), and adjusted hippocampal volume (aHV). The mediation analyses revealed that GFAP mediated the relationship between Aβ and tau deposition, and between tau deposition and reduced AD‐CM, but did not mediate the relationship between tau and aHV. Moderation analysis showed that GFAP moderated the relationship between Aβ and tau deposition.

**Conclusions:**

The findings support that astrogliosis, reflected by GFAP, mediates or moderates the key neuropathological processes related to AD.

